# Supplementary material for: Long-Term Changes in Sleep Disordered Breathing in Renal Transplant Patients: Relevance of the BMI
Source: J Clin Med. 2020 Jun 4;9(6):1739. doi: 10.3390/jcm9061739 (PMC7355565; doi:10.3390/jcm9061739)
Supplement: Supplementary file 1 [file jcm-09-01739-s001.pdf]

Journal of
*Clinical Medicine*

MDPI

*Supplementary Material*

# Long-Term Changes in Sleep Disordered Breathing in Renal Transplant Patients: Relevance of the BMI

**Francesca Mallamaci [1,2], Rocco Tripepi [1], Graziella D'Arrigo [1], Gaetana Porto [1], Maria Carmela Versace [1], Carmela Marino [1], Maria Cristina Sanguedolce [1], Giovanni Tripepi [1] and Carmine Zoccali [1,*]**

[1] CNR-IFC (National Research Council of Italy, Center of Clinical Physiology, Clinical Epidemiology of Renal Diseases and Hypertension Unit, Reggio Cal., c/o Ospedali Riuniti, Reggio Cal; francesca.mallamaci@libero.it (F.M.); rtripepi@ifc.cnr.it (R.T.); g.darrigostat@tin.it (G.D'A.); tania.porto25@hotmail.it (G.P.); permarica@gmail.com (M.C.V.); cmarino@ifc.cnr.it (C.M.); mcristinas@libero.it (M.C.S.); gtripepi@ifc.cnr.it (G.T.);

[2] Division of Nephrology and Transplantation, Department of Medicine, Ospedali Riuniti, Reggio Calabria Italy

Correspondence: carmine.zoccali@tin.it; Tel: +39-340-73540-62

**Supplemental Table S1** Concomitant therapies in the whole study population and in patients divided according to the AHI. Data are expressed as percent frequency. The total number of anti-hypertensive drugs is summarised as mean and standard deviation. All P values but the Total number of antihypertensive drugs were calculated by the Chi Square test. The P value of the number of antihypertensive drugs was calculated by ANOVA.

| | | **Baseline apnea-hypopnea index (episodes/h)** | | | |
|---|---|---|---|---|---|
| **Baseline characteristics** | **Whole group** | **<5.0** | **from 5 to < 15** | **>15** | **P** |
| | (n=221) | (n=166) | (n=37) | (n=18) | |
| Erythropoietin | 10% | 12% | 3% | 0.0% | **0.046** |
| Statins | 42% | 36% | 57% | 67% | **0.001** |
| Calcium antagonists | 49% | 46% | 46% | 78% | **0.04** |
| Beta-blockers | 51% | 51% | 54% | 44% | 0.83 |
| ACE inhibitors | 52% | 51% | 46% | 67% | 0.47 |
| Sympatholytic agents /vasodilators | 15% | 13% | 16% | 33% | **0.042** |
| Diuretics | 18% | 16% | 27% | 17% | 0.43 |
| Total number of anti-hypertensive drugs (n) | 1.85±1.16 | 1.78±1.17 | 1.89±1.07 | 2.39±1.09 | **0.046** |
| On anti-hypertensive treatment (%) | 88% | 87% | 92% | 94% | 0.23 |
| Cyclosporine (%) | 42% | 40% | 43% | 50% | 0.43 |
| Steroids (%) | 88% | 90% | 81% | 78% | **0.04** |
| Azathioprine (%) | 37% | 35% | 43% | 44% | 0.27 |
| Tacrolimus (%) | 52% | 52% | 49% | 50% | 0.72 |
| Sirolimus (%) | 4% | 4% | 5% | 0.0% | 0.71 |
| Mycophenolate(%) | 48% | 51% | 40% | 39% | 0.19 |

**Supplementary Table 2** Adjusted multiple Generalized Estimating Equations (GEE) of AHI and minimal nocturnal $O_2$ saturation over time**.** The model includes all univariate correlates of AHI at baseline with P≤0.10 (age, gender, diabetes as well as the BMI and eGFR and the change of sleep recorder Pamela→Somté. BMI and eGFR are tested as repeated measures). Data are regression coefficient (b), standard error (SE) and P value. * BMI and eGFR are introduced into the model as repeated measurements.

| | **Apnea Hypopnea Index** | **Minimal $O_2$ saturation** |
|---|---|---|
| *Variables (units of increase)* | *Regression Coefficients and 95% CI* | *Regression Coefficients and 95% CI* |
| Age (5 years) | **1,102 (1,022-,189),P=0.012** | **0,996 (0,992-,999),P=0.02** |
| Male/females | **1,841 (1,135-,984),P=0.013** | **0,979 (0,962-,997),P=0.02** |
| Diabetes (yes/no) | 1,270 (0,503-3,203),P=0.61 | 0,963 (0,904-1,026),P=0.25 |
| *BMI (1 kg/m2) | **1,109 (1,047-,174),P<0.001** | **0,993 (0,990-,996),P<0.001** |
| *eGFR (1 ml/min/1.73 m2) | 0,999 (0,989-1,009),P=0.86 | 1,000 (1,000-1,000),P=0.97 |
| Visit (0,1,2) | 1,233 (1,013-,500),P=0.037 | 0,986 (0,972-,999),P=0.047 |
| Change of the sleep recorder (yes/no) | **2,270 (1,511-,410),P<0.001** | 0,996 (0,977-1,015),P=0.68 |
